# The subjective hip value: a retrospective validation study in primary total hip arthroplasty

**DOI:** 10.1186/s42836-025-00307-0

**Published:** 2025-05-02

**Authors:** Kevin Y. Heo, Andrew Fuqua, Jason Shah, Omar Syed, Joseph Song, Emilie C. Collins, Jesse Seilern Und Aspang, Ajay Premkumar, Jacob M. Wilson

**Affiliations:** 1 Department of Orthopaedic Surgery, Emory University School of Medicine, 21 Ortho Ln, Atlanta, GA 30329 USA; 2 Department of Orthopaedic Surgery, New York Medical College, 40 Sunshine Cottage Rd, Valhalla, NY 10595 USA

**Keywords:** Patient-reported outcome measures, Subjective hip value, Total hip arthroplasty, Hip dysfunction and osteoarthritis outcome score for joint replacement, Patient outcomes

## Abstract

**Background:**

The hip dysfunction and osteoarthritis outcome score for joint replacement (HOOS JR) has been widely used to assess patient hip function. The subjective hip value (SHV) has become increasingly recognized as an efficient single-question survey for assessing hip joint function. This study aimed to determine the psychometric properties of the SHV in evaluating hip function in patients undergoing total hip arthroplasty (THA) in correlation with the traditional HOOS JR.

**Methods:**

This was a retrospective review of 1,157 distinct patients who underwent primary THA between January 2021 and December 2023. Scores for SHV and HOOS JR were collected preoperatively, as well as 3 months and 1 year postoperatively. Validity was determined using Pearson’s correlation tests between the SHV and HOOS JR.

**Results:**

Overall, the SHV was highly correlated with the HOOS JR at 3 months (R = 0.71, *P* < 0.001) and 1 year postoperatively (R = 0.79, *P* < 0.001). Additionally, changes in the SHV showed significant correlations with changes in the HOOS JR between the preoperative and postoperative periods. The SHV also had substantially fewer ceiling effects compared to the HOOS JR.

**Conclusions:**

The SHV is a valid and responsive single-item assessment for hip joint function following primary THA. Despite its limitations, its efficiency and ease of use make it a feasible option for routine clinical assessments, providing clinicians with valuable insights into patients' recovery. Subsequently, the integration of the SHV into orthopedic practice holds promise for enhancing the management of postoperative care and improving patient outcomes.

## Background

Osteoarthritis of the hip is a major contributor to decreased function and quality of life in affected individuals, often necessitating surgical intervention in the form of total hip arthroplasty (THA) [1, 2]. Given the deleterious effects associated with osteoarthritis, the primary objective of THA is the restoration of hip function, alleviation of pain, and improvement in quality of life. Consequently, effective assessment tools are crucial in the process of evaluating hip function and health status before and after surgery to appraise treatment efficacy and assist in clinical decision-making [3].

Accordingly, many validated patient-reported outcome measures (PROMs) have been constructed, each aiming to assess joint function and the effectiveness of therapeutic interventions [4–6]. Established instruments like the Hip Disability and Osteoarthritis Outcome Score (HOOS) and the modified Harris Hip Score (mHHS) are common PROMs used for evaluating hip osteoarthritis severity [7]. However, daily use of these metrics in clinical practice faces challenges due to survey length and complexity [8].

To address these issues, there has been growing interest in the use of single assessment numeric evaluation (SANE) scores in the evaluation of function in common joint pathologies [9, 10]. In particular, the subjective shoulder value (SSV) is a single-question assessment that has been widely utilized and is now established as a useful metric [11, 12]. Represented by a numeric rating from 0 to 100, the SSV is defined as a patient’s subjective shoulder assessment expressed as a percentage of a normal shoulder, with 100% representing normal joint function [11]. The SSV has been shown to offer a straightforward, easily reproducible method for assessing individuals’ subjective shoulder function and level of disability while reducing the practical constraints associated with the administration of longer, more complex PROMs [13]. Given the success of this streamlined, patient-centered approach in the evaluation of shoulder pathology, it is likely that the same metric, through the subjective hip value (SHV), can be applied to evaluating hip function and recovery after THA. One previous study has shown the SHV to be a valid and reliable assessment for patients undergoing THA in comparison to the mHHS. They alluded to increased benefits, including ease of administration and interpretability [14]. However, to the authors’ knowledge, there have not been other studies comparing the SHV to other traditional PROMs in THA.

Therefore, this study aimed to describe and assess the validity of the single-item SHV by comparing pre- and postoperative scores to the previously validated HOOS JR survey, a short-form version of the HOOS utilized for individuals undergoing THA [15]. Furthermore, the change in SHV after surgery and the change in HOOS JR scores at 3 months and 1 year were compared to analyze the sensitivity of the SHV in capturing clinically important shifts in hip function after surgery as a dynamic measure of treatment efficacy. We hypothesized that postoperative SHV is strongly correlated with postoperative HOOS JR scores, with clinically important changes seen after primary THA in both the HOOS JR and SHV scores.

## Methods

### Study and questionnaire design

Following institutional review board approval, records from patients $\ge$ 18 years of age who underwent primary THA at a single academic orthopedic center between January 2021 and December 2023 were retrospectively reviewed. Survey responses for SHV were collected, if available, at the most recent preoperative time point, as well as at the 3-month and 1-year postoperative periods. To determine the SHV, patients were asked to subjectively rate their current hip function based on the question, “What is the overall percent value of your hip if a completely normal hip joint represents 100%?”. The maximum value was 100%, and the minimum was 0%.

Expected SHV scores were also retrospectively collected, if available, at the most recent time point before surgery. Expected SHV was based on the question, “What do you expect will be the overall percent value of your hip after surgery if a completely normal hip joint represents 100%?”. Based on the collected SHV and expected SHV scores, actual and expected changes in SHV scores from the preoperative to postoperative period were then calculated. For example, the actual SHV change was defined as the actual SHV value collected during the postoperative period minus the preoperative SHV score. The expected SHV change was defined as the expected SHV collected at the preoperative period minus the corresponding preoperative SHV score.

Similar to the SHV, HOOS JR scores were retrospectively collected, if available, at the most recent preoperative time point, as well as at the 3-month and 1-year postoperative periods. To conduct the HOOS JR, patients were administered a 6-question survey incorporating specific questions about hip pain and daily function while going up the stairs, walking on uneven surfaces, rising from sitting, bending to the floor, lying in bed, or sitting down [16]. Items to each question were answered from 0 to 4, in which 0 represented no pain or loss of function, while 4 represented extreme pain or loss of function. Once the survey was completed, the sum of raw scores was converted to an interval score using a standardized table [16]. Interval score ranges were from 0 to 100, with 0 representing total hip disability and 100 representing perfect hip health.

Patients who underwent primary THA without any documented SHV or HOOS JR at any period were excluded from the study. Additionally, patients who underwent any concurrent lower extremity surgeries were also excluded from the study. Demographics, including age, sex, and BMI, were also collected. Overall, as shown in Table 1, there were 1,157 distinct patients included in the final study cohort who underwent primary THA with at least 1 survey completed at any one of the three time periods (preoperative, 3 months, 1 year). The majority of completed SHV and HOOS JR surveys were collected within the preoperative period. A potential reason for this was that, due to standard institutional protocols, patients who were signed up for surgery filled out the preoperative surveys in a clinic; however, in the postoperative period, the surveys were emailed to patients, making them less likely to be completed. As a result, of the 1,157 total patients, 182 distinct patients completed the SHV both at the preoperative period and at 3 months, while 140 patients completed the SHV at the preoperative period and at 1 year. For the HOOS JR, 182 distinct subjects completed the HOOS JR at the preoperative and 3-month period, and 148 distinct subjects completed the SHV at the preoperative and 1-year period. (Table 1).

**Table 1:** Demographic Data for Patients that Underwent Primary Total Hip Arthroplasty (THA)

Variables	Sample Size (N)
Patients completed ≥ 1 survey	1,157
Total SHV*, Pre-Op	926
Total HOOS JR*, Pre-Op	957
Total SHV, 3 months	182
Total HOOS JR, 3 months	182
Total SHV, 1 year	140
Total HOOS JR, 1 year	148
Average Age (SD*)	63.2 (12.2)
Sex (men: women) (% men)	536:621 (46.3)
BMI* (< 18.5) (%)	11 (1.4)
BMI (18.5–24.9) (%)	174 (22.7)
BMI (25–29.9) (%)	261 (34.0)
BMI (≥ 30) (%)	322 (41.9)
BMI Unknown	389

*SD s*tandard deviation, *BMI* body mass index, *SHV* subjective hip value, *HOOS JR* Hip dysfunction and osteoarthritis outcome score for joint replacement

### Data analyses

Patient demographics were assessed using descriptive statistics as shown in Table 1. The average age of patients who underwent primary THA in the study was 63.2 years of age, with around 46.3% of patients being male. For patients with a known BMI collected, the majority of patients had a BMI ≥ 30 (41.9%).

Mean SHV and HOOS JR scores were calculated at the three time points noted above, separated by the overall number of distinct subjects included in the study, as well as by those who completed all surveys at all periods. To compare the responsiveness of the SHV scores in relation to the HOOS JR, the average SHV and HOOS JR scores were plotted during the preoperative period, as well as the 3-month and 1-year postoperative periods. The average expected SHV was also determined for the preoperative period, broken down by the overall number of distinct subjects as well as the subjects that completed all surveys at all periods. At 3-month and 1-year follow-ups, the actual mean change in SHV (mean 3-month or 1-year score minus mean preoperative scores for all patients) was also calculated by the two cohorts.

Validity was determined using Pearson’s correlation tests between SHV and HOOS JR scores, per precedence [9, 17]. Correlation coefficients (R) were calculated by dividing the covariance with the product of the two variables’ standard deviations and were defined as very high (0.90 to 1.00), high (0.70 to 0.89), moderate (0.50 to 0.69), low (0.30 to 0.49), and negligible (< 0.30) [9]. Insignificant (*P*-value > 0.05) correlation coefficients were also considered negligible. Correlation coefficients represented the strength of the relationship between the SHV and HOOS JR. In the case of higher correlation coefficients, as the HOOS JR values increased, the SHV scores also increased, thus marking validity in the usage of the SHV scoring system in relation to the standard HOOS JR survey.

Thus, Pearson’s correlation tests were first applied to preoperative SHV and HOOS JR scores, then subsequently applied to postoperative SHV and HOOS JR at the 3-month and 1-year periods, respectively. Pearson’s correlation tests were also used to investigate the relationship between the change in SHV and HOOS JR scores at the corresponding postoperative periods. Finally, Pearson’s correlation tests were utilized for calculating the relationship between expected SHV change (expected SHV minus preoperative SHV) and actual SHV change (actual SHV minus preoperative SHV) for both postoperative periods. Pearson’s correlation tests were utilized for both the cohort of all distinct patients in the study, as well as the cohort of patients that completed all surveys at all three time periods. Finally, correlation tests for postoperative SHV versus postoperative HOOS JR were subsequently broken down by sex (men and women) and age group (18–49 years, 50–69, and 70+) at both the 3-month and 1-year period.

Floor and ceiling effects were also measured across both surveys and all periods. Ceiling effects occur when a considerable percentage of patients (≥ 15%–20%) score the maximum possible score (100 for both SHV and HOOS JR) [18–20], while floor effects occur when a large percentage of patients score the minimum possible score (0 for both SHV and HOOS JR). As described in Gulledge et al., high floor and ceiling effects suggest an inability for the PROM to differentiate among those at the lowest and highest ends of the spectrum, respectively [17].

Finally, the minimum clinically important difference (MCID) was calculated for the SHV and HOOS JR at 1 year. The MCID is a threshold value of change in a PROM score that is deemed to have clinical significance [21]. It can be used as a tool for determining the effectiveness of a particular treatment. The MCID was calculated by multiplying the standard deviation of the preoperative SHV scores by 0.50, as previously described [22]. This threshold value was then utilized to identify the percentage of patients that had a value for the difference between the 1-year SHV and the preoperative SHV greater than the MCID threshold. All data analyses for this study were performed using R Studio version 4.2.3 (PBC, Boston, Massachusetts).

## Results

### Baseline outcomes

**Fig. 1:**
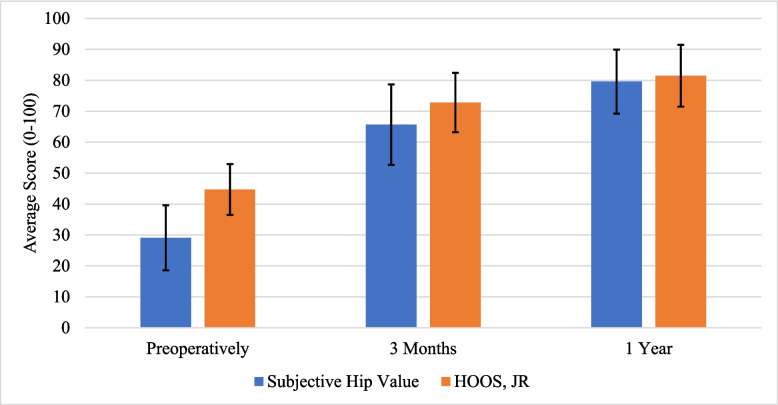
Chart illustrating changes in subjective hip value (SHV) and Hip Dysfunction and Osteoarthritis Outcome Scores for Joint Replacement (HOOS JR) scores from the preoperative study period to the 1-year postoperative period

Average values for SHV, expected SHV, and HOOS JR during the preoperative period are shown in Table 2. On average, patients had an SHV of 29.1 preoperatively but were expected to have an average SHV of 90.4 after primary THA. Average values of SHV, change in SHV, and HOOS JR at the 3-month and 1-year periods are also shown in Table 2. Both the SHV and HOOS JR had an increase in scores at each postoperative period from baseline. The SHV increased on average by 37.2 at the 3 months, and by 49.7 at the 1 year. The time course of the function is shown in Fig. 1.

**Table 2:** Baseline Data and Hip Score Measurements Before and After Primary Total Hip Arthroplasty (THA)

Variables	All Distinct Subjects	Subjects that Completed All Surveys
	Mean (SD)	Mean (SD)
SHV, Pre-Op	29.1 (21.0)	35.8 (25.2)
HOOS JR, Pre-Op	44.7 (16.4)	43.5 (17.1)
Expected Post-Op SHV	90.4 (13.9)	92.3 (24.4)
SHV, Post-Op, 3 months	65.7 (26.0)	72.9 (22.4)
SHV Change, 3 months	37.2 (30.8)	37.1 (34.6)
HOOS JR, Post-Op, 3 months	72.8 (19.2)	76.7 (18.0)
SHV, Post-Op, 1 Year	79.6 (20.7)	80.0 (23.8)
SHV Change, 1 Year	49.7 (27.3)	44.2 (37.5)
HOOS JR, Post-Op, 1 Year	81.5 (20.0)	78.8 (21.6)

*SD* standard deviation, *SHV* subjective hip value, *HOOS JR* Hip Dysfunction and Osteoarthritis Outcome Score for Joint Replacement

For patients who completed both the SHV and HOOS JR at all periods, patients had an SHV of 35.8 preoperatively, but were expected to have an average SHV of 92.3 after primary THA. Furthermore, they had an average HOOS JR of 43.5 preoperatively. The average SHV was 72.9 at the 3 months and 80.0 at the 1 year, and the average HOOS JR was 76.7 at the 3 months and 78.8 at the 1 year (Table 2).

### Correlation between SHV and HOOS JR at 3 months

Preoperative SHV and HOOS JR scores demonstrated a low correlation (R = 0.43, *P* < 0.001; Table 3), while postoperative SHV and HOOS JR scores at 3 months demonstrated a high correlation (R = 0.71, *P* < 0.001). Thus, in the 3-month postoperative period, there was a remarkably strong relationship in which the greater the HOOS JR score, the greater the SHV score. Pearson’s correlation tests indicated a moderate relationship between the change in SHV scores and the change in HOOS JR scores at 3 months (R = 0.64, *P* < 0.001). Furthermore, the expected change in SHV and the actual change in SHV demonstrated a high correlation at 3 months (R = 0.71, *P* < 0.001).

**Table 3:** Correlation Between Subjective Hip Value (SHV) and Outcome Measures Before and After Primary Total Hip Arthroplasty (THA)–3 Months

All Distinct Subjects	Correlation Coefficient (R)	Coefficient of Determination (R^2^)	*P*-value	Relationship
Pre-Op SHV vs. Pre-Op HOOS	0.43	0.18	< 0.001	Low
Post-Op SHV vs. Post-Op HOOS	0.71	0.50	< 0.001	High
Men Only	0.72	0.52	< 0.001	High
Women Only	0.69	0.48	< 0.001	Moderate
18–49 Years Old	0.71	0.51	< 0.001	High
50–69 Years Old	0.75	0.56	< 0.001	High
70 + Years Old	0.61	0.37	< 0.001	Moderate
SHV Change vs. HOOS Change	0.64	0.41	< 0.001	Moderate
Expected SHV Change vs. Actual SHV Change	0.71	0.50	< 0.001	High
**Subjects that Completed All Surveys**	**Correlation Coefficient (R)**	**Coefficient of Determination (R**^**2**^**)**	***P*-value**	**Relationship**
Pre-Op SHV vs. Pre-Op HOOS	0.43	0.18	0.04	Low
Post-Op SHV vs. Post-Op HOOS	0.78	0.61	< 0.001	High
SHV Change vs. HOOS Change	0.78	0.61	< 0.001	High
Expected SHV Change vs. Actual SHV Change	0.87	0.76	< 0.001	High

HOOS JR (HOOS) = Hip Dysfunction and Osteoarthritis Outcome Score for Joint Replacement

The SHV and HOOS JR at the 3-month postoperative period also demonstrated significant correlations across all demographic subgroups. The relationship was high among men (R = 0.72, *P* < 0.001), patients aged 18–49 years old (R = 0.71, *P* < 0.001), and patients aged 50–69 years old (R = 0.75, *P* < 0.001).

For patients who completed the SHV and HOOS JR at all periods, preoperative SHV and HOOS JR scores similarly demonstrated a low correlation (R = 0.43, *P* = 0.04), while postoperative SHV and HOOS JR scores at 3 months similarly demonstrated a high correlation (R = 0.78, *P* < 0.001). Pearson’s correlation tests indicated a high relationship between the change in SHV scores and the change in HOOS JR scores at 3 months for these subjects (R = 0.78, *P* < 0.001). The expected change in SHV and the actual change in SHV also demonstrated a high correlation at 3 months (R = 0.87, *P* < 0.001).

### Correlation between SHV and HOOS JR at 1-Year

During the 1-year postoperative SHV and HOOS JR scores demonstrated a high correlation (R = 0.79, *P* < 0.001; Table 4). As a result, in the 1-year postoperative period, there was a remarkably strong relationship in which patients who reported a greater HOOS JR score also reported a greater SHV score. Pearson’s correlation tests indicated a moderate relationship between the change in SHV scores and the change in HOOS JR scores at 1-year (R = 0.64, *P* < 0.001). The expected change in SHV and actual change in SHV demonstrated a significant correlation in the 1 year (R = 0.71, *P* < 0.001).

**Table 4:** Correlations Between Subjective Hip Value (SHV) and Outcome Measures Before and After Primary Total Hip Arthroplasty (THA)–1 Year

All Distinct Subjects	Correlation Coefficient (R)	Coefficient of Determination (R^2^)	*P*-value	Relationship
Post-Op SHV vs. Post-Op HOOS	0.79	0.62	< 0.001	High
Men Only	0.82	0.67	< 0.001	High
Women Only	0.77	0.59	< 0.001	High
18–49 Years Old	0.67	0.45	0.004	Moderate
50–69 Years Old	0.83	0.69	< 0.001	High
70 + Years Old	0.81	0.66	< 0.001	High
SHV Change vs. HOOS Change	0.64	0.41	< 0.001	Moderate
Expected SHV Change vs. Actual SHV Change	0.71	0.50	< 0.001	High
**Subjects that Completed All Surveys**	**Correlation Coefficient (R)**	**Coefficient of Determination (R**^**2**^**)**	***P*-value**	**Relationship**
Post-Op SHV vs. Post-Op HOOS	0.85	0.72	< 0.001	High
SHV Change vs. HOOS Change	0.63	0.40	0.004	Moderate
Expected SHV Change vs. Actual SHV Change	0.77	0.59	< 0.001	High

*HOOS JR (HOOS)* Hip Dysfunction and Osteoarthritis Outcome Score for Joint Replacement

The SHV and HOOS JR during the 1-year postoperative period also demonstrated a significant correlation across all demographic subgroups, as shown in Table 4. The relationship was high among men (R = 0.82, *P* < 0.001), women (R = 0.77, *P* < 0.001), patients aged 50–69 years old (R = 0.83, *P* < 0.001), and patients aged 70 + years old (R = 0.81, *P* < 0.001).

For the patients who completed the SHV and HOOS JR at all periods, postoperative SHV and HOOS JR scores at 1 year similarly demonstrated a high correlation (R = 0.85, *P* < 0.001). Pearson’s correlation tests indicated a moderate relationship between the change in SHV scores and the change in HOOS JR scores at 1 year for this cohort (R = 0.63, *P* = 0.004). The expected change in SHV and the actual change in SHV similarly demonstrated a high correlation at 1 year (R = 0.77, *P* < 0.001).

### Floor and ceiling effects

Table 5 shows the calculated floor and ceiling effects for the SHV and HOOS JR at all three time periods. Floor effects were comparable between the two PROMs at all periods. However, ceiling effects were greater among the HOOS JR at the 3-month and 1-year postoperative periods. At 3 months, 13.7% of patients with a completed HOOS JR had a maximum score, while 1.5% of patients with a completed SHV had a maximum score. During the 1 year, 34.8% with a completed HOOS JR had a maximum score, while 4.7% of patients with a completed SHV had a maximum score.

**Table 5:** Floor and Ceiling Effects of the Subjective Hip Value (SHV) and the Hip Dysfunction and Osteoarthritis Outcome Score for Joint Replacement (HOOS JR)

Timepoint	Floor Effect in %		Ceiling Effect in %	
	**SHV**	**HOOS JR**	**SHV**	**HOOS JR**
**Preoperative**	2.7	2.5	< 1.0	1.1
**3 months**	< 1.0	0	1.5	13.7
**1 Year**	0	< 1.0	4.7	34.8

*SHV *subjective hip value, *HOOS JR * Hip Dysfunction and Osteoarthritis Outcome Score for Joint Replacement

### Minimum clinically important difference

The standard deviation and derived MCID threshold for the SHV and HOOS JR at the preoperative period are shown in Table 6. Overall, 82.7% of patients showed a relevant improvement in hip function at 1 year by exceeding the MCID measured using the SHV, while 85.9% of patients exceeded the MCID measured using the HOOS JR.

**Table 6:** Standard Deviation and Calculated Minimal Clinically Important Differences (MCIDs) for the Subjective Hip Value (SHV) and the Hip Dysfunction and Osteoarthritis Outcome Score for Joint Replacement (HOOS JR) at 1 year

Score	Standard Deviation (SD)a	MCID (SD × 0.5)	Percent of Patients Exceeded MCID (%)
SHV	21.0	10.5	82.7
HOOS JR	16.4	8.2	85.9

*SHV *subjective hip value, *HOOS JR *Hip Dysfunction and Osteoarthritis Outcome Score for Joint Replacement

astandard deviation of preoperative baseline values

## Discussion

In this study, we demonstrated the validity and responsiveness of a single-item SHV as a measure of postoperative function and recovery at 3 months and 1 year following primary THA, finding that SHV scores were highly correlated with HOOS JR scores at 3 months and 1 year postoperatively. Additionally, the SHV score provided a valid assessment of clinically significant changes in joint function following THA with fewer ceiling effects compared to the HOOS JR and achieved a similar MCID to the HOOS JR. This study adds further evidence supporting the utility of SHV for evaluating the outcomes of THA [23].

The use of PROMs has become an increasingly relevant factor in clinical decision-making and outcomes-related research. SANE scores have continued to gain traction as a potential alternative due to their simplicity, efficiency, and ease of administration in comparison to the HOOS JR [24]. Despite the simple nature of these metrics, recent data have confirmed their validity in evaluating knee, shoulder, spine, and elbow function after surgery [9, 11, 14, 25–27]. Currently, however, there is a paucity of evidence examining the validity of the single-item subjective hip value (SHV) score for postoperative evaluation of primary THA outcomes. In a comparison of the SHV against the modified Harris Hip Score (mHHS), Leopold et al. found SHV to be a valid and reliable tool for analyzing hip function after primary THA for hip OA [14]. Additionally, comparable ceiling and floor effects were noted, as well as achievement of minimal clinically important differences needed to evaluate changes in function after surgery. Similarly, we found SHV scores to be highly correlated with HOOS JR scores at both 3 months and 1 year after primary THA, with this trend holding consistent across sex and age groups. Additionally, correlations of moderate strength between changes in SHV and changes in HOOS JR scores before and after surgery were noted, with a majority of the surveyed population achieving a minimal clinically significant change from baseline score with both PROMs.

Floor and ceiling effects are a limitation noted with some PROMs and refer to the inability of a survey to capture further deterioration in health status below a minimum score or further improvement above a maximum score [28, 29]. While Leopold et al. found the SHV to have comparable floor and ceiling effects to the mHHS, we demonstrated that SHV was less susceptible to the occurrence of ceiling effects than the HOOS JR following THA at 1 year [14]. This may be partially explained by the fact that patients are less likely to rate their overall hip status with extremely low or high values, leaving room for noted improvement or decline in function over time with SHV assessments. Furthermore, in high-functioning patients, a perfect score on the HOOS JR is relatively easier to achieve, whereas the SHV is adapted to each patient based on their expectations. With improving outcomes after THA, demands in hip function are likely to surpass simple activities such as climbing stairs or putting on shoes, as people want to return to sports and other strenuous activities. Therefore, patients can individualize their activity and expected functions with the SHV, which potentially accounts for the lower ceiling effects. Overall, these results suggest the potential utility of the SHV for the assessment of hip status during long-term follow-up, although further research at longer follow-up periods is needed.

While there are several advantages of utilizing the SHV after THA, the use of SHV still carries inherent limitations that must be acknowledged. For example, the SHV has increased susceptibility to subjectivity and a lack of nuance related to individual assessments of pain, function, or other quality-of-life measures available in multi-item assessment tools. Gilbart et al. showed that the subjective shoulder value (SSV) was a valid assessment tool after surgery for different pathologies, including rotator cuff tears, glenohumeral osteoarthritis, and glenohumeral anterior instability; however, they also demonstrated that there was not one clinical variable that could completely predict the SSV [11]. Thus, in the context of THA, utilization of the SHV may make it potentially difficult for clinicians to understand specific components during the postoperative period that need to be addressed or treated.

This study possesses several key strengths. Consistent with prior evidence, our data demonstrated the validity of SHV in assessing clinically significant changes in hip function after THA. Additionally, the use of the HOOS JR as a validating model for the SHV offers distinct advantages over the modified Harris Hip Score (mHHS), as it provides a more comprehensive evaluation of pain, function, and quality of life [15, 30]. This further strengthens the quality of evidence asserting the utility of SHV for regular clinical use to track changes in global hip health after primary THA.

Nevertheless, there are important potential limitations to consider when interpreting this study. There was a substantial lack of available postoperative surveys among patients with baseline preoperative survey scores. Although the reasons for this are likely multifactorial, one potential explanation is due to institutional standard protocols in which preoperative surveys were given to patients during their clinical visit after signing up for surgery. Meanwhile, in the postoperative period, surveys were emailed to patients, thus making them less likely to be completed. This may have introduced nonresponse bias into the study; however, similar values of pre- and postoperative HOOS JR and SHV scores and high correlation coefficients were shown between the two PROMs utilizing the patients who completed both surveys at all periods. Nevertheless, prospective methods are needed in the future to control for potential loss of follow-up and strengthen the overall power of the study. Potential strategies to mitigate the loss of follow-up in future studies may include administering surveys during postoperative visits, directly calling patients to encourage survey completion, integrating electronic survey reminders via portal messages, or offering incentives for participation. We also did not assess the viability of the SHV for long-term follow-up past one year postoperatively, although the low occurrence of ceiling effects may suggest a unique role for the SHV in long-term postoperative follow-up. Future studies should consider investigating the long-term validity of the SHV after THA, as utilization of the SHV long-term may provide direct clinical insights into changes in postoperative activities, pain, or function.

## Conclusions

In conclusion, this study provides supporting evidence to demonstrate the validity of the SHV in assessing global hip function following primary THA. Due to the ease of administration and ability to detect clinically significant changes in hip function, with lower ceiling effects than other commonly used PROMs, the use of the SHV has several potential benefits in the assessment of hip arthroplasty patients. Overall, future prospective research is needed to understand the full effects of utilizing the SHV in patients undergoing THA.

## Data Availability

The datasets used and/or analyzed during the current study are available from the corresponding author on reasonable request.
